# A qualitative analysis of the information science needs of public health researchers in an academic setting

**DOI:** 10.5195/jmla.2018.316

**Published:** 2018-04-01

**Authors:** Shanda L. Hunt, Caitlin J. Bakker

**Affiliations:** Health Sciences Libraries, University of Minnesota, 305 Diehl Hall, 505 Essex Street SE, Minneapolis, MN 55455; Health Sciences Libraries, University of Minnesota–Twin Cities

## Abstract

**Objectives:**

The University of Minnesota (UMN) Health Sciences Libraries conducted a needs assessment of public health researchers as part of a multi-institutional study led by Ithaka S+R. The aims of the study were to capture the evolving needs, opportunities, and challenges of public health researchers in the current environment and provide actionable recommendations. This paper reports on the data collected at the UMN site.

**Methods:**

Participants (n=24) were recruited through convenience sampling. One-on-one interviews, held November 2016 to January 2017, were audio-recorded. Qualitative analyses were conducted using NVivo 11 Pro and were based on the principles of grounded theory.

**Results:**

The data revealed that a broad range of skill levels among participants (e.g., literature searching) and areas of misunderstanding (e.g., current publishing landscape, open access options). Overall, data management was an afterthought. Few participants were fully aware of the breadth of librarian knowledge and skill sets, although many did express a desire for further skill development in information science.

**Conclusions:**

Libraries can engage more public health researchers by utilizing targeted and individualized marketing regarding services. We can promote open science by educating researchers on publication realities and enhancing our data visualization skills. Libraries might take an institution-wide leadership role on matters of data management and data policy compliance. Finally, as team science emerges as a research priority, we can offer our networking expertise. These support services may reduce the stresses that public health researchers feel in the current research environment.

## INTRODUCTION

Scientific research is in a stage of transition. Popular media can be skeptical about the validity of scientific research results [1–4], skepticism that is coupled with rapidly changing technologies that both challenge and advance current scientific methods [5–7]. Perhaps most notably, economic resources for academic research are declining, with the National Science Foundation reporting that university research and development has experienced the longest multiyear decline in funding since 1972 [8].

These recent shifts have been documented as challenges to the field of public health [9–12]. A diverse, multidisciplinary field—defined as “the combination of science, skill and beliefs that is directed to the maintenance and improvement of the health of all people through collective and social action” [13]—public health grapples with the changing research environment, a balance between community and academic engagement, and the need to communicate and collaborate across disciplines. The Centers for Disease Control and Prevention (CDC) have called for innovation in data collection, integration, analysis, and dissemination and a reimagining of how public health information is communicated to various audiences [14].

While this sounds challenging, academic libraries are in a place to partner with public health researchers during this transitional period. Libraries are in a process of perpetual modernization, “moving from a focus on daily production and a model of reactive service, to a proactive stance that has broader institutional impact” [15]. New models of service provision have emerged, including the informationist model, functional specialists, and hybrid positions [16, 17]. As the models through which services are delivered become more agile, libraries have been able to expand their traditional offerings to incorporate support for data management, knowledge synthesis, translational research, and digital scholarship [18, 19].

There is ample opportunity for partnerships between academic libraries and public health researchers, but first, there must be an understanding of researchers’ current information practices and beliefs. The information needs and uses of public health practitioners and workers—those employed by government or nonprofit agencies—have been examined thoroughly [20–23]. However, few studies have addressed the needs of public health faculty researchers in an academic setting, which can differ given the resource-rich environment that universities offer. Curtis, Weller, and Hurd found that public health faculty researchers did not take full advantage of the training opportunities that libraries provided [24], and Wallis found that they infrequently engaged with librarians and library services in their information-seeking activities despite being regular users of information [25].

While these studies provide an important baseline, they were conducted in 1997 and 2006 and do not reflect the context of modern academic research environments or library services. A recent systematized review added to the literature by examining studies that included public health workers in academia, private organizations, government departments, and clinical settings, but the review focused on evidence-based practice rather than all-inclusive library services and did not report findings according to work environment [26]. The current study assessed the comprehensive information science needs and behaviors of public health research faculty in the School of Public Health (SPH) at the University of Minnesota (UMN).

### Setting

The UMN SPH is one of the first public health programs offered in the United States and is ranked 8th in the nation [27, 28]. SPH offers 21 master’s degrees and 4 doctoral programs, and includes more than 130 faculty in 4 divisions: biostatistics, environmental health sciences, epidemiology and community health, and health policy and management [29].

UMN libraries employ a functional specialist model to support the work of liaisons and highly specialized collaboratives to build capacity throughout the library system. In addition to established librarian activities in instruction, reference, and collection development, we have developed extensive support for research services. We support research data management through workshops, consultations, and an institutional data repository. We are strong advocates of open access models, as evidenced through our open access author fund, our support for complying with the National Institutes of Health (NIH) public access policy, and our publishing services department. We have further expanded into areas of research impact assessment, as seen in our Policy & News Media Impact Service and Experts@Minnesota, a research networking system. Finally, we provide consultations and workshops on grant funding and management of one’s online academic identity.

## METHODS

This qualitative needs assessment was part of a larger multi-institutional study coordinated by Ithaka S+R. The study aimed to understand the specific needs of public health researchers and provide actionable recommendations to better serve them. This paper reflects the data collected at UMN. UMN’s Institutional Review Board (IRB) determined that this project did not qualify as human subjects research and did not require IRB review.

### Participants

Participants were recruited through convenience sampling. The recruitment list was downloaded from the UMN SPH list of faculty members. Eighty faculty were contacted via email, and twenty-four agreed to participate (Table 1).

**Table 1:** Distribution of interview participants

Department	Assistant professor	Associate professor	Professor	Total n (%)	School of Public Health (SPH) total* (%)
Biostatistics	2	1	1	4 (17%)	18%
Environmental health	1	2	3	6 (25%)	20%
Epidemiology and community health	0	4	5	9 (38%)	40%
Health policy and management	1	1	3	5 (21%)	23%
Total n (%)	4 (17%)	8 (33%)	12 (50%)		
SPH total* (%)	18%	38%	45%		

* SPH total refers to noncontract faculty employed by the SPH.

### Procedure

Participants agreed to one-hour interviews with one of the investigators. There was no incentive. Interviews were collected from November 2016 to January 2017, digitally audio-recorded, and lasted nineteen to seventy-one minutes. Recordings were supplemented with field notes regarding additional conversations and general impressions. Audio files were sent to a professional online transcription company.

### Measure

The semi-structured interview instrument was developed by Ithaka S+R in consultation with Medical Library Association and American Public Health Association leadership. The UMN investigators made small revisions to the eleven-item questionnaire, restructuring some questions and adding a question on researcher self-promotion, resulting in a sixteen-item questionnaire (supplemental appendix).

### Data analysis

Qualitative analyses were conducted using NVivo 11 Pro [30] and were based on the principles of grounded theory [31]. Both authors independently coded 4 interviews using line-by-line open coding, after which coding agreement was assessed and a coding scheme developed. Transcripts and field notes were independently coded by the authors, and the 2 NVivo databases were merged. NVivo’s coding comparison found 84%–100% agreement between coders on all codes. The discrepancy can be explained by the fact that the first author coded large sections of text, while the second author coded smaller sections of the same text. Finally, the authors pulled emergent themes from the data by documenting relationships among codes, identifying recurring concepts, and relying on their own existing knowledge on the topics—ending in a theoretical framework [31].

## RESULTS

The interviews provided insight into the participants’ workflows. Their research areas spanned a wealth of topics, from tobacco use to hydraulic fracturing. They often worked on more than one unique research project at a time (up to seven). Their educational backgrounds and varied collaborations highlighted the interdisciplinary nature of public health, with participants identifying forty-eight disciplines ranging from psychology to civil engineering to political science.

Relationships with potential collaborators developed organically at meetings, conferences, and forums, and participants relied on word-of-mouth to find new collaborators. Partnerships were formed with researchers from their own divisions, the SPH, departments across UMN, and other universities, in order of frequency. Participants also frequently partnered with local and national health departments as well as local and state agencies. Less frequently, they partnered with community members.

Participants shared details about their research activities, culminating in six distinct themes: (1) participants were comfortable with their current literature search and citation management strategies, (2) data management was an afterthought, (3) participants misunderstood the publication industry, (4) participants highly valued dissemination in many forms, (5) participants viewed professional marketing as distasteful, and (6) participants believed challenges in the field outweighed opportunities. Their activities and needs around these topics are presented here with supporting evidence and quotes. Direct quotations have been edited for readability.

### Participants were comfortable with their current literature search and citation management strategies

Participants sought various types of information throughout the research life cycle. The frequency of literature searching depended on their familiarity with a topic. Some participants had been working in the same research area for decades, so they relied on their existing knowledge. Although one researcher was familiar with subject headings, most engaged in more passive information retrieval activities, such as relying on electronic tables of contents or suggestions from Google Scholar profiles. Less than a handful of participants discussed systematic search strategies.

Participants utilized a variety of sources to meet their information needs. PubMed and Google Scholar were both frequently referenced, as were general search engines like Google. Some participants also utilized subscription databases such as SciFinder, Ovid MEDLINE, and PsycINFO.

In addition to literature, participants searched for datasets, health statistics data, measurement instruments (e.g., surveys), and statistical and research design methodologies. Participants placed a high value on grey literature and accessed it in a variety of ways:

I know there’s great enthusiasm for looking into grey literature. Not just unpublished documents, dissertations, things that might’ve been presented at conferences—but not published…looking at social media, looking at what’s on the web.

Sources included community partners, funding agencies, health organizations, national archives, Wikipedia, and social media. Some did speak of the difficulties in knowing how and where to look for unpublished information, especially in terms of searching for data sources.

When asked what kinds of challenges they faced in information seeking, most participants said not being able to access a specific article. Some were aware of interlibrary loan (ILL), and others asked colleagues at other institutions to access it for them. Most, however, said they simply gave up.

Once participants obtained the information that they sought, most organized it using a citation manager, with EndNote and Mendeley being the most popular. A significant number of participants used their own literature filing systems: some created their own databases using a tool like Microsoft Excel, one used email, and another stated: “I think I’m using the desktop.” No one reported being completely satisfied with any of their organization methods.

Participants expressed a desire and willingness to learn more, given the opportunity and time. Several participants had sought one-on-one help from a librarian in developing search strategies and using library resources.

### Data management was an afterthought

Few participants worked entirely with qualitative or quantitative data. Their research data often challenged strict definitions of data types: one participant who worked with animal models referred to her data as both “quantitative” and “observational.” Spreadsheets were the most commonly used format. Tools for analysis were more diverse, with participants referencing SAS, R, SPSS, MATLAB, and NVivo. The need to use multiple tools was recognized: “I have to use regularly three or four or five in my everyday life because none of them are complete.” The responsibility of data management fell on different research team members: data manager (written into the grant), coordinating center, data analysis team, or research coordinator.

A combination of storage media was used: university or departmental servers, personal computers, flash drives, Microsoft Access (even though the tool is not meant for long-term data storage [32]), NetFiles (UMN resource no longer in existence), Dropbox, and Google Drive. Large, multisite projects utilized more sophisticated strategies, featuring coordinating centers and distributed databases. International collaborations were seen as particularly complicated, as the data needed to be contained in a certain geographic location or were subjected to additional security considerations.

Sharing data throughout the analysis process was a more complicated issue. At times, they used storage tools for sharing (e.g., Google Drive), but other times they transferred files via file transfer protocol (FTP). Email was the most common way to share projects. Administrative assistants sometimes shared data on behalf of senior faculty participants, who were, as a result, unsure what methods were used in that process. While data sharing was generally viewed positively, some challenges were noted:

[I]t’s very hard to keep up with where those datasets are and whether you’ve shared them with people who might still have them. And even if it’s requested to get it back, did they respond to your email? Did you follow up? So that’s part of interdisciplinary work, sharing data…I worry about it and it’s a big issue, who to share with and whether they’re going to protect it the way I do, particularly if they’re at another institution.

Participants also noted broadly sharing datasets underlying study results. Most who had shared datasets in the past had done so to satisfy funding or journal requirements or at the individual request of another researcher. A few had shared to promote research reproducibility or because someone else on the research team had decided to do so. One participant noted that data sharing was a smooth process that forced them to concretely wrap up the project, while another noted that it was time consuming.

Those who had never shared their underlying data expressed that they did not think anyone would care to see the data, had concerns over how the data would be used in the future (e.g., informed consent restrictions and ability of others to interpret the data), or were open to it as long as controls were in place to screen users of the data:

I haven’t considered it; I’d be very hesitant to do so because if people don’t have the training to analyze that data and interpret it correctly—I know there can be different ways of interpreting data, but at least interpreting it within the realm of what would be considered correct or supported—then that data can…cause harm, eventually. What I would be open to is if there was a trusted resource or repository where those individuals evaluate requests for data, and grant access to data, a limited subset of the data, based on a review of whether the people have the qualifications.

There were concerns about intellectual property and data sharing, with one participant referring to “a whole cadre of people whose only job is pilfering other people’s stuff, or parasitically using it.” Not all participants who were unfamiliar with data sharing felt negatively about it: a few felt it was important for students to have access to real datasets for training purposes, and others felt that data should be available to taxpayers and the communities being researched.

Participants frequently kept their research data indefinitely. When they indicated that they destroyed data, the timeline for destruction was ambiguous. While the data were retained, they were rarely revisited or reanalyzed. The retention was largely precautionary: “[T]here’s always this slight anxiety that someday someone will ask me ‘how did you get that result?’” Several participants equated data storage to “hoarding,” while others felt data were “invaluable.”

When speaking about data, participants acknowledged the importance of documentation. They felt that their documentation was often insufficient, causing them to misplace or distrust old data, or they indicated that they did not have the necessary time and resources. Participants who searched for secondary data sets found insufficient documentation frustrating: data do not have value if they cannot be discovered.

Participants reported a number of problems around data management. They lost data due to the discontinuation of their storage tools, had old data stored on zip or floppy disks, had staff take data with them when they left, or had data that was not documented and, therefore, unusable. Even if participants had not personally experienced data loss or access issues, several expressed concern over losing data in the future.

### Participants misunderstood the publication industry

Participants preferred traditional publication methods such as peer-reviewed research articles in journals that were considered “top tier,” had high impact factors, reached desired audiences, and published related articles. Journal selection was connected to the existing incentive system: faculty relied on publication in particular journals to achieve promotion and tenure. Secondary factors in selecting a journal included perceived innovation of the article, turnaround time, and word limit.

Participants had mixed feelings about impact factor as a measurement of journal quality and its value in the journal selection process. Many subdisciplines in public health, such as health policy and health services, are not well served by impact factor, leading to skepticism about its helpfulness in the journal selection process. However, in more well-served subdisciplines, such as toxicology, it was seen as a proxy for quality.

While participants felt comfortable with traditional publishing, they identified some limitations. The different submission requirements for each journal were described as “cumbersome and unpleasant,” and one participant described the challenge of lengthy review processes:

I have a paper right now that has been in revision for three years, just rounds and rounds of revision. There’s nothing fundamentally wrong with the paper, it’s just they’re picky. But then once this does get published—and it is a good journal that it will be published in—I don’t even care anymore ’cause it’s been so long. And no one cares anymore ’cause it’s an old idea by now.

Most participants had submitted at least one article to an open access journal, although someone else on their research team might have decided to do so. There were mixed impressions: some were very happy with the review process and production; others had concerns regarding the quality of peer review, relaxed standards for acceptance, and potential negative implications for promotion and tenure. Participants were aware of predatory journals but did not feel they could identify them.

Participants were deterred by article processing charges (APCs), describing them as “insulting” and “hilarious.” Those who had paid APCs had done so using discretionary, grant, division, and start-up funds. One faculty member put aside money they earned as a consultant for the purpose of paying APCs.

While publishing in open access journals was divisive, a few faculty mentioned making their preprints available through repositories, particularly bepress and arXiv. A few participants also talked about publishing in a traditional journal, but then making the portable document format (PDF) file of that article available on their faculty, division, or personal website in order to reach a broader audience. Whether their publication agreements allowed this was not clear.

Participants misunderstood the funding structures of both open access and traditional publishers, assuming that open access publishers were commercialized, while traditional publishers were not. One participant believed that traditional publishers made their money with membership fees. One faculty described themself as philosophically opposed to APCs:

[W]hen journals start charging you to publish your work, I just feel like there’s a commercialism aspect to it that I feel runs counter to…the system in which we do research. Almost all of our research is funded, externally funded…And I don’t like it if people suddenly start trying to make money off that. I don’t know that it’s a good use of taxpayer money, to be able to pay publishers to put my work out there when there are options to not do that.

### Participants highly valued dissemination in many forms

Most participants valued disseminating their research findings beyond articles, and they did so via multiple avenues. Most commonly, they distributed their work through presentations at conferences, seminars, and webinars or in reports to funders, various stakeholders, and community partners. At times, their outputs were variations of publications, such as book chapters or white papers, and other times, they were for the purpose of getting information out to the public, in newsletters and policy briefs. A few participants were statisticians and felt that sharing their code via repositories was an important part of their work.

Participants identified three emerging avenues through which to disseminate their work to broader audiences: data sharing (discussed above), social media (discussed below), and data visualization. Participants wanted to be able to “tell a story” in a few images that would be easily understood and interpreted. There was a spectrum of comfort with this concept. Some were attempting to create visuals with PowerPoint, Excel, Word, and Photoshop, but most indicated that their creations were basic and they needed support. Participants who were more engaged in this area, generally methodologists and statisticians, utilized programs such as MATLAB, SigmaPlot, and R but were self-taught in their visualizations.

Despite the great interest in data visualization, there were concerns that the subtle nuances of research could not be summarized in an infographic or that the visual representation would drown out the message. Investing in data visualization might also require a tradeoff: “One of the questions I ask people when I’m giving talks on writing and publishing is, ‘Are you better off to hire an analyst or a graphic artist?’” Whereas resources were once allocated to gathering and analyzing data, researchers are now considering redistributing those resources to support more effective communication of data, rather than its analysis.

### Participants viewed professional marketing as distasteful

Participants largely denied involvement in self-promotion, with only one participant being fully in favor of researchers marketing themselves. Many expressed discomfort with the concept, finding it “distasteful.” They often felt that professional marketing was embedded in their publication activities. One participant stated that researchers who marketed themselves were not doing quality work. And yet, they reported that they felt pressure from their divisions to broadly market themselves, and the divisions and the SPH often did so on their behalf.

A few participants engaged in self-promotion by keeping their faculty web pages updated. They mentioned having profiles on LinkedIn or ResearchGate, but none put effort into maintaining those profiles. While participants were generally intrigued by the possibilities that popular social media (i.e., Twitter) afforded, they wondered about who would manage such a campaign and what resources would be required. Some participants articulated possible benefits of self-promotion, imagining that it could open doors to new collaborations, give them a leg-up in a competitive field, or provide publicity that could lead to funding opportunities.

### Participants believed challenges in the field outweighed opportunities

Participants were asked about both challenges and opportunities in their field, and they spoke about them in tandem. Almost unanimously, funding was identified as the biggest challenge. Waning resources have led to a competitive funding environment and reduced support staff, with high turnover rates, less time to produce quality publications, and conflicting obligations. Nearly all participants cited a lack of time as a barrier. One faculty member described their desire to attend events where they could learn about emerging topics and technologies:

That sounds interesting, but I’ve got 120 papers to grade by tonight. I don’t know if everybody faces that, but I know they face deadlines for getting grant proposals in and reports in and that what you would prefer to do loses out to what you have to do…It’s not a luxurious job in classic academia where you teach a seminar to ten students once a week and sit in your office and think the rest of the time. That’s the opposite. This place is a factory. And if you don’t manage your time, you fall behind. Lose your job.

While time and money were most frequently discussed, participants shared additional challenges for the field. They felt that people did not see the value of public health and that there has not been enough communication with the public regarding the benefits of the absence of disease. They identified opportunities in educating the public on how research agendas are set, highlighting the cost effectiveness of research, and including community members as active participants in research:

I think we could be more intentional about how we engage with members of the community, broadly defined—not just individuals and families, but organizations, nonprofit organizations, departments of health, industries—in how we set research agendas, so that what we’re doing is going to actually be viewed as valuable by stakeholders within the broader community.

[I]f societies understood the importance of prevention and its cost effectiveness truly, they’d be incredibly more productive and they’d have tons of money to spend on something other than a fancy new stent to put in my fourth heart attack. It’s crazy the way we allocate resources for healthcare. And somebody’s got to change that—so it’s got to be us.

Participants stated that, as a result of this disconnect, the uptake of proven interventions has been poor and policy recommendations have been ignored. They cited cost, lack of a clear communication mechanism, and potential for misinterpretation as barriers to communicating with the public. They also worried that the public might feel overwhelmed by constant messaging. And yet, there were a few strong opinions that this work is the responsibility of public health researchers.

Beyond greater community engagement, participants saw the need for new partnerships that could prove to be challenging. One participant felt that public health academics tended to reject economic theories, yet several participants stated there must be more cost/benefit analyses in the future of public health. A number of participants also foresaw the need to partner with corporate entities, which they anticipated would be counterintuitive to most public health researchers. At the same time, they were excited by the upcoming trends in team science that could enable them to engage in new research relationships.

## DISCUSSION

Libraries are persistent and innovative in addressing user needs by offering workshops, integrated instruction, sophisticated tools, and specialized training. These worthwhile efforts should continue, while libraries simultaneously imagine new ways to understand and address user needs. This paper offers unconventional solutions that sometimes borrow from other disciplines such as business and psychology.

### Target and individualize support for information retrieval

Participants had established approaches to locating and organizing information and were generally comfortable with their strategies, but comfort does not necessarily signify proficiency. They expressed anxiety that their searches might miss key articles and confusion regarding which resources to use when they searched grey literature. Overall, the literature organization strategies they described were cumbersome and required dedicated time.

Given the inherent interdisciplinary nature of public health, participants relied heavily on grey literature, personal awareness, and search strategies such as pearling (i.e., finding literature by searching the body or reference list of an article). Beahler et al. found pearling to be a common strategy in public health due to the range of resources required and incomplete indexing in biomedical databases [33]. Reliance on grey literature may also be rooted in the unique information needs that are not met by traditional published articles (e.g., data and statistics, evaluations of existing community programs, organizational recommendations, etc.) [26]. Similar to how public health practitioners working in different contexts have different information needs [34], the breadth of topics and scale of work—ranging from individual interventions to population-level studies—require a diverse range of information types. Public health as a discipline may need more in-depth training on grey literature search strategies than other professions.

Participants displayed a lack of awareness of existing library services, including ILL and one-on-one consultations. That researchers may not be aware or take full advantage of library services and training opportunities is a common theme [25, 26, 35, 36]. Coupled with the extensive and diversified information needs and lack of time to dedicate to new activities, public health faculty are perhaps best served with individualized training, be that within divisions (e.g., environmental health, biostatistics), research areas (e.g., cardiovascular disease, alcohol policy), professional status (e.g., assistant professor, full professor), or one-on-one. A report on individualized learning of medical students claimed that this method opened dialogue between trainer and trainee, allowed for flexibility in time constraints, and helped trainees prioritize skills [37]. This method could benefit the librarian (trainer)/faculty (trainee) relationship on all points. Specifically, the ability for faculty to prioritize the skill sets they desire to learn would be essential to their investment in the learning process. Dreyfus’s model of adult skill acquisition requires that the learner stay emotionally involved to reach the stage of competence (stage three) [38].

Librarians must also consider how best practices in marketing can inform communication of such services. In targeted marketing, specific subsets of users are identified and serve as the focus of marketing efforts, and those efforts concentrate on defining and describing products and services from the unique perspective of that subset. Such targeted messaging is significantly more likely to be favorably received by the intended audience and to create a perception of value [39]. These subsets should be sufficiently granular (i.e., “services for assistant professors in epidemiology” rather than “services for faculty”) and carefully evaluated.

### Take the lead on data management

A systematic review on barriers to data sharing in public health identified several data management errors as the reason for inefficient sharing [40]. Many of those errors were named by participants in this study as well. Overall, data management was an afterthought for participants. They used ineffective storage solutions and had unclear archiving protocols. Despite the frequency of collaboration, no one mentioned the use of a collaboration tool, such as Box or Open Science Framework. Most participants named at least one aspect of data management that was significant to them, but they did not grasp the breadth of data management or its long-term importance. This was not surprising given the pressures they felt to simply complete their research with the time and resources allotted.

Beyond data management training, which is now common practice in libraries [19, 41], there is opportunity for a solution that is more embedded in university processes. As funder, publisher, and government policies develop and require researchers to report on data management specifics, it is imperative for both researchers and their institutions that individuals comply. One way to achieve compliance is through institutional policies. At some European universities, this model of high-level strategic planning has helped researchers understand data management activities, regulations, and mechanisms [41]. These policies can serve to highlight the resources that are available to researchers throughout an institution—including the university libraries, office of the vice president for research, and IRB—in order to streamline the compliance process.

Participants were not well versed in practices for sharing data with broad audiences, and, consistent with Saunders et al.’s findings [42], they had concerns about giving up control. They seemed to be unaware of IRB permissions related to data sharing, embargos, or licenses requiring author attribution. Many of the expressed concerns can be alleviated by these mechanisms. Moreover, the conversation surrounding data sharing is a nuanced one. Not all data are suitable for public dissemination. Open, frank discussion of what data can and should be shared, and in what format and forum, is essential to meeting the CDC’s goals of maximizing access to research data, while simultaneously protecting privacy and confidentiality [14]. Again, with support and buy-in from high-level research groups within a university, mass education could be mandated or encouraged.

The top-down approach to systemic change requires a culture shift. Asking researchers to meticulously manage data throughout the research life cycle with the possible end purpose of data sharing is another task for which researchers do not have time, and a top-down approach to organizational change may be at odds with the established attitudes and practices of researchers. Katzenbach, Steffen, and Kronley have established five principles for successful culture shift in an organization, one of which is to focus on a few critical shifts in behavior [43]. This step requires a “safe space,” where trusted allies can motivate people and model best practices. Librarians are in an ideal position to fulfill this role as they have established, supportive relationships with many faculty members and frequently mentor university community members.

### Promote open science by improving comprehension of publication structures

It was apparent that participants did not see publishing in open access journals as a legitimate alternative to publishing via more traditional methods, possibly due to a misunderstanding of the funding structures of both. While most traditional publishers have profit margins between 20% and 30% [44, 45], participants did not recognize that these traditional publishing models were, in fact, commercial models. Participants expressed discomfort with APCs and believed that an author-pays model created a commercial enterprise.

Lack of funds for APCs is a known impediment to publishing both in gold open access and hybrid journals [46, 47]. A gold journal is one that is fully open access, with every article available, while a hybrid journal is one that allows individual authors to make specific articles open access in a journal that would otherwise be subscription based. It should be noted that APCs for hybrid journals are consistently higher than those for gold journals, with recent studies reporting hybrid journal APCs to be between $1,200 and $1,300 USD more expensive than their gold journal counterparts [48–50]. Participants were not aware of the distinction between hybrid and gold journals or the associated cost implications. While researchers should not be expected to have an in-depth understanding of the publishing industry, grasping certain aspects is useful in navigating the publishing landscape and can encourage broader dissemination.

Challenges in understanding business models extended to licensing and rights. Several participants did engage in green open access, or self-archiving, through repositories and personal websites. However, participants did not consider publication agreements when making those materials available. One responsibility of libraries is to educate researchers about what can and cannot be legally shared and to encourage researchers to negotiate with publishers to ensure they retain sufficient rights to meet their personal objectives. This includes providing expertise in understanding publication agreements, author addenda, institutional open access policies, and copyright and fair use. Broader dissemination encourages greater transparency and a more robust scholarly discussion [51], and, as such, increasing understanding of author rights and sharing options can inspire researchers to further facilitate open science.

### Support communication of public health practices to broad audiences

The issue of communicating public health to the public arose as a challenge and, at times, was not viewed with importance. Not only does the CDC name this as a priority [14], but it is essential in translating research into practice given that public health practitioners working in nonprofit organizations often have more limited access to library resources than public health academics [20, 21], a point that that participants did not mention. Public health practitioners are part of “the public,” and they require current research results to successfully intervene with their target populations.

Data visualization is increasingly recognized in public health as a mechanism for knowledge mobilization, the process of moving research into active use in policy and practice [52, 53]. Participants were deeply interested in data visualization, but they also expressed concerns about how to visualize data effectively, particularly how to use visualization for health messaging without oversimplifying research findings. This is a relatively new and emerging area for libraries, many of which are determining how these services integrate with and extend libraries’ core services [54]. Librarians should continue to advance their data visualization knowledge and skill sets, and, libraries, at a minimum, should continue to act as networking hubs to connect researchers with other resources on campus that can assist them, ranging from informatics or communications departments to statistical support.

In speaking of self-promotion, participants framed activities in terms of public relations rather than in terms of science communication. Self-promotion as a tool of communicating one’s work broadly and its potential connections to translational science were overlooked by participants. The efficacy of self-promotional acts can be controversial. In organizational psychology, studies have found that self-promotion is effective in creating perceptions of competence, but overt acts of self-promotion make an individual appear arrogant [55–57]. Beyond navigating this balance between projecting confidence and arrogance, when researchers’ self-promotion is disconnected from science communication, it may be seen as being at odds with the research endeavor [58]. Despite these opposing considerations, self-promotion is, at its core, research promotion. A social media strategist recently wrote about the importance of research promotion as a way to engage students, bring media attention to academic research, and gain a competitive edge [59].

While researcher self-image is beyond the scope of library services, there are possibilities for knowledge mobilization. Activities could include developing plain language summaries or infographics of research outputs and leveraging institutional repositories to distribute them. Librarians can also engage in conversations with researchers about the ways in which networking tools and social media are being used to communicate with the public.

### Provide a bridge between funding and collaboration

Finally, although there is a limit to the assistance a library can provide in helping public health researchers overcome their funding challenges, there are a few key areas for partnership. Funding is a primary concern, but some of the expressed concerns might have been alleviated by the recent news that Congress has approved an additional $2 billion to the NIH budget [60]. Even so, these concerns are longstanding. Librarians are in a position to help researchers explore ways of locating grant and foundation funding. We can also support researchers with their time and monetary resource deficit by assisting in many aspects of the grant submission process: literature reviews, data management plans, submission tools, and public access policy compliance. The information specialist model was developed to provide support in this manner by acting as an expert embedded in a research team and advising on best practices throughout the life cycle [16]. To consider the process of acquiring and maintaining funding as a component of the research life cycle is a logical extension of this role.

While participants did not point to their collaborations as areas for opportunity, we see them as such. Collaborations are the backbone of team science, an opportunity that participants identified. The data presented here point to engagement in interdisciplinary research, yet these results were, in part, skewed due to the participants’ training and background. Most held several degrees, and their past training was not public health, per se, so their personal diversity brought an innate interdisciplinary nature to any research team. They also put great emphasis on collaborations as an important part of their work, yet they most often collaborated with individuals who were part of their own divisions or one of the three other divisions in the SPH. It was much rarer for participants to collaborate with researchers from other departments and universities.

The lack of true interdisciplinary collaborations could affect research funding in the SPH. A recent study found that research team innovation and efficiency improved due to the risk and resource sharing that happens with team science. They found that established collaborations attracted more funding, even more so than publication productivity [61]. Academic libraries are uniquely positioned to offer support in this area. The library is an institutional hub for information, and liaison librarians who have personal connections across the university are particularly well positioned to assist in making networking connections. Knapp suggested that libraries and librarians can offer social introductions, physical space, interdisciplinary literature searching, cross-discipline vocabulary, and collection development [62]. Additionally, tools such as faculty profiling systems create opportunities for discovery and collaboration.

### Limitations

This study provides a snapshot of the public health research faculty at UMN, a large and well-established research institution, which is not generalizable to all public health researchers at other universities. In fact, there is controversy around the generalizability of qualitative research studies in general. Instead, this study offers high-level, in-depth information that can inform future programming and research, and the multi-institutional report published by Ithaka S+R will provide a better picture of the practices and needs of a more representative sample. Whereas in the SPH, full professors represented 45% of noncontract faculty, they represented 50% of our sample. Conversely, associate professors were slightly underrepresented at 33%, in comparison to 38% of all noncontract faculty in SPH. In a few short years, a similar study may yield different answers, as full professors retire and junior faculty move into more prominent roles. We would anticipate that a future sample would be more open to the changing landscape surrounding research in general, particularly in the areas of data management and open science.

## CONCLUSION

The participants were experienced researchers. There was a broad range of skill levels, particularly in the area of information retrieval, and there were areas of misunderstanding and confusion, as seen in the researchers’ assessment of the current publishing landscape and open access. Data management emerged as an area for education opportunity. Few participants were fully aware of the depth and breadth of librarian knowledge and skill sets, although many did express a desire for further skill development in information science. Librarians can engage more public health researchers by utilizing targeted and individualized marketing regarding services such as grey literature searching and support for researcher promotion. We can promote open science by educating researchers on publication realities and enhancing our data visualization skills. Librarians might take an institution-wide leadership role on matters of data management and data policy compliance. Finally, as team science emerges as a research priority, we can offer our networking expertise. These support services may reduce the stresses that public health researchers feel in the current research environment.

## SUPPLEMENTAL FILE

Appendix Interview instrument
