# Supplementary material for: A qualitative analysis of the information science needs of public health researchers in an academic setting
Source: J Med Libr Assoc. 2018 Apr 1;106(2):184–97. doi: 10.5195/jmla.2018.316 (PMC5886501; doi:10.5195/jmla.2018.316)
Supplement: Appendix [file jmla-106-184-s001.pdf]

JMLA Journal of the Medical Library Association

SC

Supplemental content to
J Med Libr Assoc. Apr;106(2):dx.doi.org/10.5195/jmla.2018.316
www.jmla.mlanet.org
© Hunt, Bakker 2018

A qualitative analysis of the information science needs of public health researchers in an academic setting

Shanda L. Hunt; Caitlin J. Bakker, AHIP

APPENDIX

Interview instrument

1. Describe your current research focus or projects.
   a. In what ways is your work interdisciplinary?

2. What research methods do you currently use to conduct your research?
   a. Are these methods typical for your field?
   b. Do you collaborate with others as part of your research?
      i. Explain. (Who, division of work, how information is created and stored)

3. What kinds of data does your research typically generate?
   a. Tell me what your research data outputs look like.
   b. Do you think of how to present your data visually?

4. How do you manage and store your data?
   a. For what purpose do you store your data?
   b. For how long do you store your data?
   c. Do you ever return to your old data for any reason?

5. When you are formulating a new research idea, what kinds of information do you rely on when doing your literature review?
   a. How do you locate this information?

6. How does information retrieval differ at the initial stages of research versus during the implementation or analysis phase?
   a. How do you manage and store information for your ongoing use?
   b. Do you experience any challenges with information retrieval?

7. Think about a past or ongoing research project where you faced challenges in researching the initial idea. Describe these challenges.
   a. What could have been done to mitigate these challenges?
   b. Are there any other challenges you regularly experience when conducting research?

8. More broadly, what do you think the trends in public health research are?
   a. How do you keep up with trends?

9. Where do you typically publish your research in terms of the kinds of publications and disciplines?
   a. What factors are important to you when choosing a journal?
   b. Do you disseminate your research beyond scholarly publications? Where?
      i. Why do you choose to publish via this venue?
   c. Are your publishing practices typical for public health researchers? Explain.

Supplemental content to
J Med Libr Assoc. Apr;106(2):dx.doi.org/10.5195/jmla.2018.316
www.jmla.mlanet.org
© Hunt, Bakker 2018

10. Have you ever made your publications available through open access?
    a. If yes, what was your motivation for doing so?
        i. What was the overall experience like for you?
    b. If no, why not?

11. Have you ever made your research data or materials available through open access?
    a. If yes, where did you make them available?
        i. What was your motivation for doing so?
        ii. What was the overall experience like for you?
        iii. Was there a charge? How was the cost paid for?
    b. If no, why not?

12. Throughout your career, how have you marketed yourself as a researcher?
    a. Did you consciously think about how marketing yourself and dissemination of your work are linked?

13. What future challenges do you see for the broader field of public health?

14. What future opportunities do you see for the broader field of public health?

15. If you were to imagine any services or tools that could make your research and publication process smoother, what would that look like?

16. Is there anything else about your experiences as a public health scholar that you think is important for me to know that was not covered in the previous questions?
